# A Review of Ultrasonic Treatment in Mineral Flotation: Mechanism and Recent Development

**DOI:** 10.3390/molecules29091984

**Published:** 2024-04-25

**Authors:** Huan Zhang, Mingming Du, Haijie Hu, Hongli Zhang, Naijian Song

**Affiliations:** 1 College of Chemistry and Material, Weinan Normal University, Weinan 714099, China; zhongl@wnu.edu.cn (H.Z.); snaijian@wnu.edu.cn (N.S.); 2 State Key Laboratory of Multiphase Flow in Power Engineering (SKLMF), Xi’an Jiaotong University, Xi’an 710049, China; 4120103181@stu.xjtu.edu.cn; 3 Shaanxi Province Key Laboratory of Environmental Pollution Control and Reservoir Protection Technology of Oilfields, College of Chemistry and Chemical Engineering, Xi’an Shiyou University, Xi’an 710065, China; 21211070888@stumail.xsyu.edu.cn

**Keywords:** ultrasonic, mineral flotation, flotation agents, slurry

## Abstract

Ultrasonic treatment has been widely used in the mineral flotation process due to its advantages in terms of operational simplicity, no secondary pollutant formation, and safety. Currently, many studies have reported the effect of ultrasonic treatment on mineral flotation and shown excellent flotation performance. In this review, the ultrasonic mechanisms are classified into three types: the transient cavitation effect, stable cavitation effect, and acoustic radiation force effect. The effect of the main ultrasonic parameters, including ultrasonic power and ultrasonic frequency, on mineral flotation are discussed. This review highlights the uses of the application of ultrasonic treatment in minerals (such as the cleaning effect, ultrasonic corrosion, and desulfuration), flotation agents (such as dispersion and emulsification and change in properties and microstructure of pharmaceutical solution), and slurry (such formation of microbubbles and coalescence). Additionally, this review discusses the challenges and prospects of using ultrasonic approaches for mineral flotation. The findings demonstrate that the application of the ultrasonic effect yields diverse impacts on flotation, thereby enabling the regulation of flotation behavior through various treatment methods to enhance flotation indices and achieve the desired objectives.

## 1. Introduction

**Figure 1:**
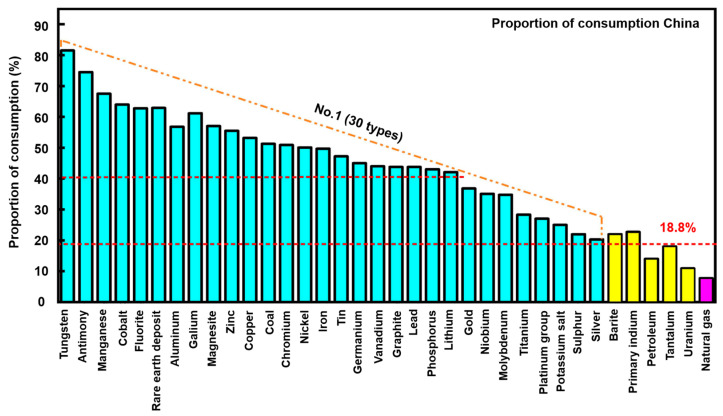
Proportions of mineral resources consumed in China in 2018 [1,3].

Mineral resources are important material bases for economic and social development [1]. With the gradual progress of global industrialization, especially in developing countries, the global demand for mineral resources is expected to continue to grow rapidly in the coming decades. At present, the situation of supply and demand of mineral resources in China is challenging [2]. In the short term, the situation where per capita resources are lower than the world level is difficult to change. The percentage of consumption of mineral resources in China in 2018 is displayed in Figure 1 [1,3]. In this figure, the consumption of 30 of the 36 minerals is shown to be over the world average; among them, 22 minerals account for more than 40% of the total global consumption, and the latter 6 minerals receive an average consumption of 18.8% [3]. Given today’s depletion of high-quality ores and rich resources, the importance of economic and environmentally friendly beneficiation treatment for low-grade and complex iron ore has become increasingly prominent [4,5,6]. Thus, the mineral processing industry requires efficient treatment processes and advanced technical support in order to effectively and selectively separate valuable minerals from gangues.

**Figure 2:**
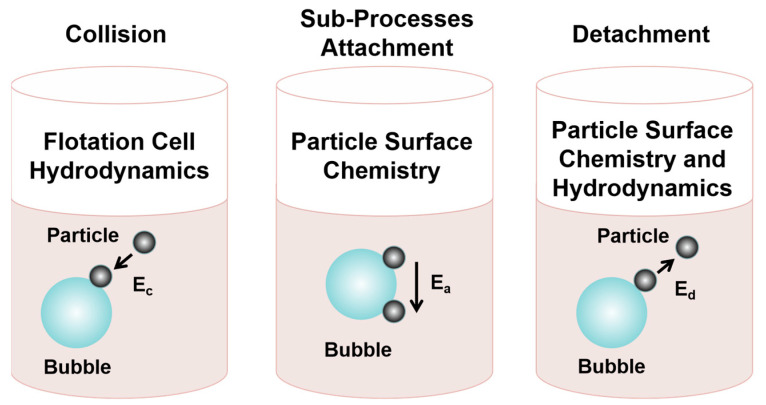
Sub-processes of the interaction between a particle and a bubble in flotation [20].

**Figure 3:**
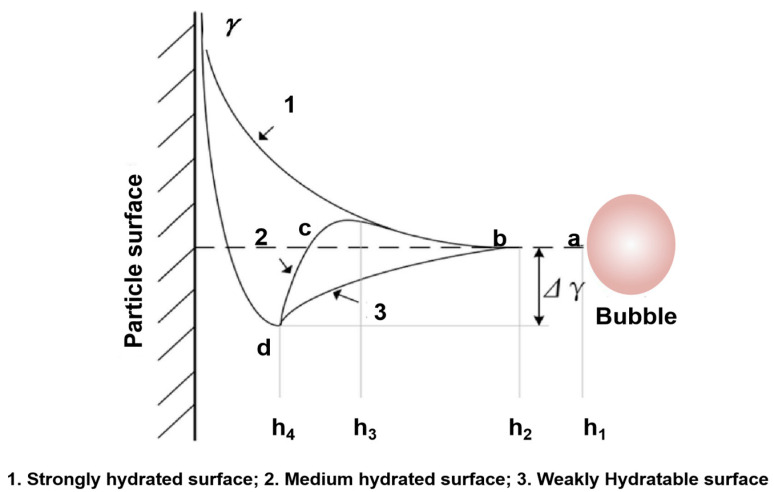
Changes in the thickness and energy of a hydration film [3].

In the 19th century, flotation was the most innovative and efficient mining technique, used to process over two billion tons of mineral resources per year [7,8]. Flotation is a process that uses bubbles to separate particles, which is commonly used in mineral processing [9,10], wastewater treatment [11], paper deinking [12], oil sand processing [13], grease recycling [14], microbial separation [15], and plastic recycling [16]. Flotation is based on the differences in the surface hydrophobicities of different materials [17,18,19]. The interaction process of particles and bubbles is viewed as consisting of three sub-processes, namely, collision, attachment, and detachment [20,21], as shown in Figure 2. Together, these three factors determine the collection probability of particles trapped by bubbles. In order for hydrophobic particles to be successfully captured by bubbles, particles and bubbles must first be close enough, that is, particles and bubbles must collide effectively, which is usually determined by the hydrodynamics of the flow field where particles and bubbles are located [21]. Secondly, the hydration film between the hydrophobic particles and the bubbles becomes thin and breaks, and a three-phase contact periphery begins to form and reaches a stable state, that is, the particles adhere to the surface of the bubbles and form particle–bubble aggregates [22]. Finally, when the external force exerted on the particles is greater than the force between the particles and the bubbles, the particles will break away from the surfaces of the bubbles. When the external force is less than the force between the particles and the bubbles, the particles will be successfully recovered by flotation [23]. It is worth noting that the particle–bubble adhesion process is relatively complex. As the particles approach the bubbles, a hydration layer forms between the water dipoles and between the water molecules and the particle surfaces. Due to the extrusion of bubbles, the hydration layer becomes thinner. An energy barrier forms as the hydration layer thins, and only when the energy barrier is crossed will the hydration layer break. In order to induce collision contact between particles and bubbles, an external environment is needed to perform the task of rapidly breaking the hydration layer. Subsequently, particles and bubbles begin to come together spontaneously, and as the contact surface gradually increases, particles adhere stably to the surfaces of the bubbles [24]. When the bubbles begin to come close to the surfaces of different hydrophobic particles, the energy changes, as shown in Figure 3 [3]. The size range of the mineral in the flotation process must be about 20–150 μm to achieve a satisfactory flotation effect. Generally, the optimal flotation size range is affected by factors such as flotation unit type [25], bubble size [26], particle size [27,28], froth stability/thickness [26], reagent [29], turbulence intensity [30,31], etc. In this technique, components such as particles, oil droplets, contaminants, etc., are separated from the mixture based on their hydrophilic or hydrophobic surface properties [32,33,34]. In order to improve the flotation performance of these difficult-to-treat coals, various types of chemical [35,36,37,38], physical [39,40,41], and physico-chemical [42,43,44,45] beneficiation techniques are commonly employed. Factors such as the fine mineral size, water entrainment, slime coating, and surface oxidation may affect the efficiency of these processes, leading to flotation results that commonly do not meet industrial requirements [46,47,48]. To overcome these problems, ultrasonic technology has been studied for many years for its potential to improve flotation efficiency [49,50,51]. However, our basic understanding of how ultrasonic treatment affects flotation is still limited.

In general, ultrasonic treatment involves the use of acoustic energy to treat solutions, suspensions, and solid materials in liquids. When the frequency is higher than 20 kHz, it is considered an ultrasonic wave. An exciting feature is that ultrasonic treatment is a green process, and it can be used for medicine, environmental governance, pharmaceutics, food processing, and surface cleaning [52,53,54]. In terms of mineral flotation, ultrasonic treatment under appropriate conditions can improve flotation efficiency [55,56,57]. A large number of studies have shown that under the action of strong cavitation and fluidization, the incorporation of ultrasonic treatment can significantly improve the process efficiency with a shorter treatment time, lower energy consumption, and lower reagent consumption [58,59,60,61]. Most of the mechanical effects of ultrasonic treatment contribute to emulsification, cleaning, dispersion, and fragmentation [62]. The adsorption of the flotation collector and the floatability of minerals can be changed through the cleaning and cracking effect of ultrasonic treatment on a mineral surface [63,64]. The chemical effect of ultrasonic cavitation is in favor of the desulfurization of high-sulfur coal. In addition, the influence of ultrasonic cavitation on bubbles or foam layers cannot be ignored. So, it is necessary to systematically summarize the application of ultrasonic treatment in all aspects of mineral flotation.

The primary objective of this review is to provide an overview of the effects of ultrasonic treatment on mineral flotation. Accordingly, the present review covers the following: (1) a brief introduction to the ultrasonic mechanism; (2) a discussion on the effects of the main ultrasonic parameters (ultrasonic power and ultrasonic frequency) on mineral flotation; (3) a systematic summary of the effects of the application of ultrasonic treatment on mineral flotation; and (4) the research direction and future research prospects.

## 2. Ultrasonic Mechanism

**Figure 4:**
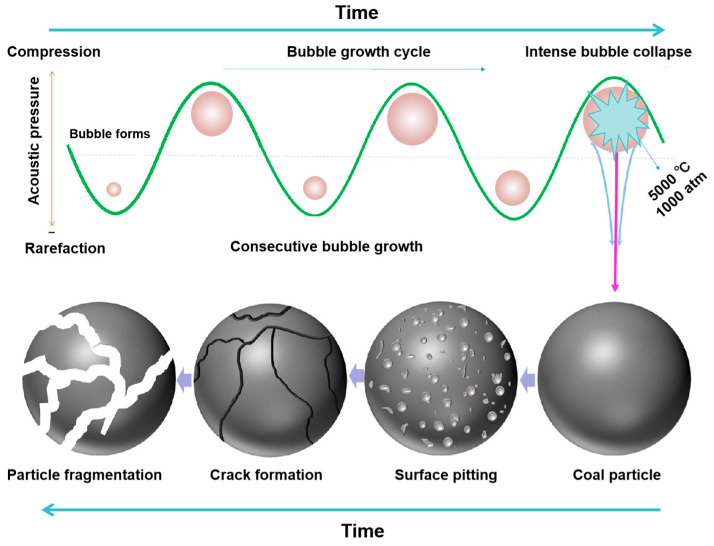
Ultrasonic mechanism of coal processing [65].

The application of ultrasonic treatment in coal processing has been a hot research field for decades. The principle of ultrasonic technology applied to flotation is mainly based on the cavitation effect and acoustic radiation effect of liquid in ultrasonic treatment. In the process of acoustic cavitation, ultrasonic activation destroys the attractive forces of molecules in the solution, so the aqueous medium undergoes alternating cycles of thinning and compression under the influence of ultrasonic vibration, as shown in Figure 4 [65]. When the energy of the sound field reaches the cavitation threshold, the cavitation bubbles will close and collapse [66]. Ultrasonic cavitation can cause basic effects such as a thermal effect, mechanical effect, and chemical effect.

### 2.1. Ultrasonic Cavitation Effect

Ultrasonic cavitation includes a transient cavitation effect and stable cavitation effect [67]. Different cavitation states depend on the sound pressure and sound frequency. The two kinds of cavitation phenomena can exist simultaneously in a medium. Under certain conditions, steady-state cavitation can be transformed into transient cavitation. Transient space-time and steady-state cavitation can work together on mineral flotation.

#### 2.1.1. Transient Cavitation Effect

It is generally believed that transient cavitation can only occur under the action of large sound intensity and can only exist for one or up to several sonic cycles. When the sound pressure amplitude is greater than the static pressure in the liquid, the initial radius of the bubble is smaller than the resonance radius [68], due to the arrival of the negative-pressure phase of sound pressure, and when the positive-pressure phase of sound pressure arrives, the bubble first continues to increase to the maximum half-diameter. Then, it rapidly shrinks until it collapses and closes. This closed bubble movement is often called “instantaneous space-time” [69]. This process generally requires a large sound intensity (greater than 10 W/cm^2^) and a short life cycle of cavitation bubbles. In the process of sound field oscillation and growth, the bubble will continuously accumulate energy. When the bubble is completely closed (collapsed), the accumulated energy inside the bubble will be released in the form of a shock wave radiating outward, thus producing an effect on the surrounding materials.

**Figure 5:**
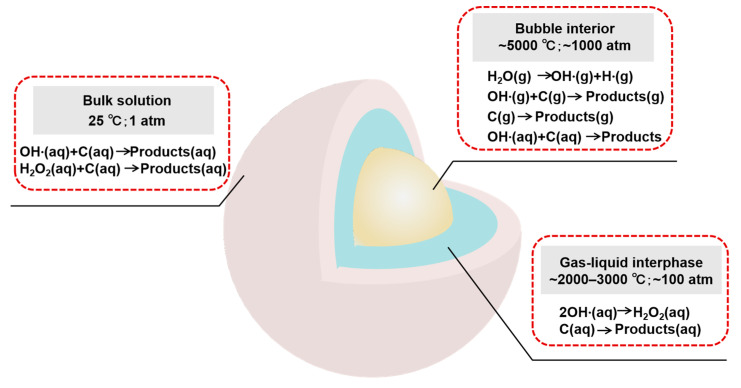
Acoustic cavitation in water.

The high temperature (~5000 °C) and high pressure (~1000 atm) associated with transient cavitation can provide a physical basis for explaining the generation of free radicals, supercritical water, and sonoluminescence phenomena [70,71,72] (Figure 5). The free radicals can combine with each other and then produce a series of reactions [73]. This effect, known as the chemical effect of ultrasonic treatment, has been widely used in chemical synthesis [74], the food industry [75], wastewater treatment [76], etc. The high pressure is explained as the direct cause of increasing molecular collision and increasing chemical reaction activity.

**Figure 6:**
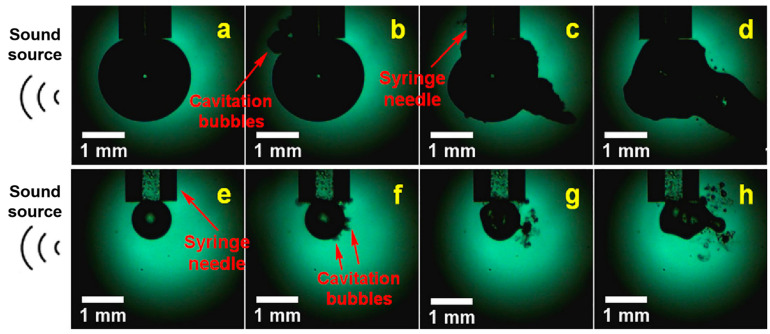
Transient cavitation effect on a large bubble ((**a**) 0 μs; (**b**) 20 μs; (**c**) 120 μs; (**d**) 600 μs) and a kerosene droplet ((**e**) 0 μs; (**f**), 20 μs; (**g**) 120 μs; (**h**) 1 ms) [65].

Transient cavitation collapses violently produce many effects. It is generally believed that the characteristics of transient cavitation are as follows: first, there is an obvious threshold value to generate transient cavitation. Because the cavitation effect of transient cavitation is generally much stronger than that of a steady state, in many cases, transient cavitation is the main consideration. Therefore, the transient cavitation threshold plays an extremely important role in the measurement of the cavitation effect. Second, the difference between transient cavitation and stable cavitation is that the former collapses and generates local high temperature and pressure, also known as a hotspot. From the energy point of view, the bubble absorbs the energy of the sound wave during the resonance process, and when the bubble collapses, the energy is concentrated in a very small area, so although the energy density is low in a large range, the energy density can be extremely high in a small local area [77]. The transient cavitation effect on a large bubble can be seen in Figure 6.

**Figure 7:**
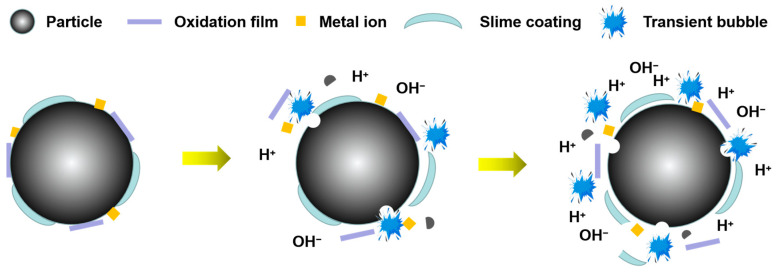
Effect of ultrasonic transient cavitation on mineral flotation [78].

The huge energy released by the bubble in the transient cavitation process will have a strong impact and stripping effect on incompatibilities of the mineral surface, so that the impurities fall off in the aqueous solution, and the mineral will be exposed to a fresh surface, which is conducive to mineral flotation. In addition, transient cavitation also has a dissolution effect on mineral surface components, exposing them to active sites, which is also conducive to flotation, as shown in Figure 7.

#### 2.1.2. Stable Cavitation Effect

When the sound pressure is much smaller than the static pressure in the liquid, under the action of weak sound field, the stable small-amplitude pulsation phenomenon produced by the bubbles is usually called “stable cavitation”. The stable cavitation bubbles will continue to oscillate without collapsing [79]. The stable cavitation effect is the effect of the formation of tiny bubbles by nuclei [66]. The existence of tiny bubbles is conducive to improving the collision efficiency with fine particles [80,81].

**Figure 8:**
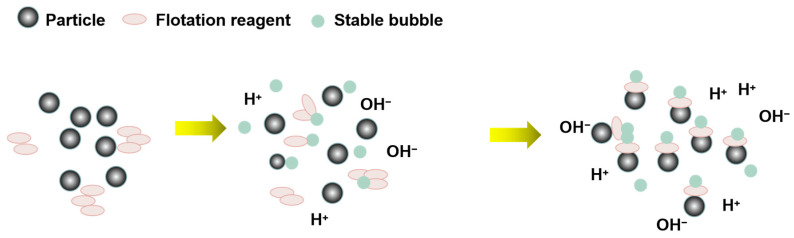
Effect of ultrasonic stable cavitation on mineral flotation [78].

Stable cavitation can dissolve the contaminants on the surface of the object. The stable cavitation bubbles form between the surface of the object and the contaminated layer, and they oscillate to dissolve the pollutants in the foam. Therefore, stable cavitation can be used to remove soluble impurities from the mineral surface and disperse and emulsify flotation agents (Figure 8). Stable cavitation also generates a large number of stable tiny bubbles, which oscillate frequently. In this process, the surface activator has enough time to adsorb on the bubble/solution interface and dissolve more gases to form bubbles, giving full play to its effect [82]. Microbubbles enhance particle aggregation through bridging, thus increasing the collision adhesion probability between particle aggregates and flotation bubbles and improving mineral floatability [81,83].

### 2.2. Acoustic Radiation Force Effect

**Figure 9:**
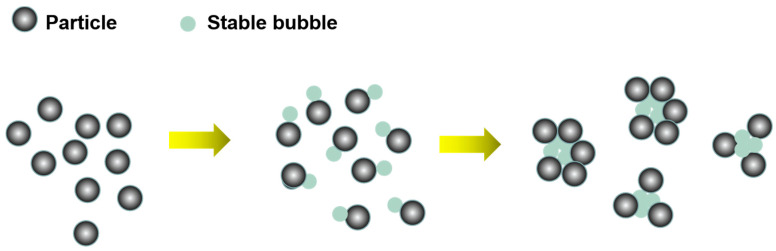
Effect of acoustic radiation force on mineral flotation [78].

The acoustic radiation force effect is related to the movement of bubbles or particles. In addition to the movement related to the cavitation effect, bubbles will also aggregate in a certain external environment and form a bubble group in a certain shape [84], perhaps the “cluster of grape clusters” [85], “cluster of cone bubbles” [86], or “Lichtenberg diagram” [87]. Thus, the flotation bubbles obtain a large carrying capacity with which to collect minerals (Figure 9). Under the action of the acoustic radiation force effect, these bubbles containing fine-grained minerals can aggregate those minerals through polymerization, thus improving the effective adhesion rate between minerals and bubbles [66].

## 3. Effect of Main Ultrasonic Parameters on Mineral Flotation

### 3.1. Ultrasonic Power

**Figure 10:**
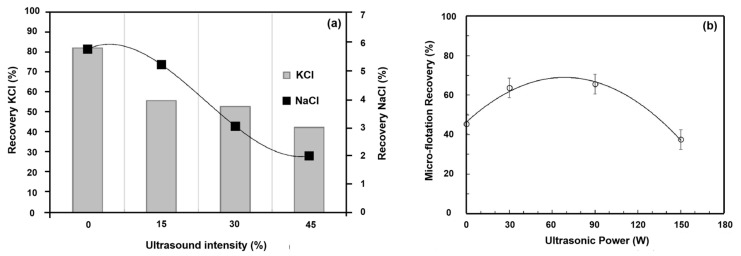
Effect of the ultrasound intensity (**a**) [50] and the ultrasonic power (**b**) [89] on the flotation results.

**Figure 11:**
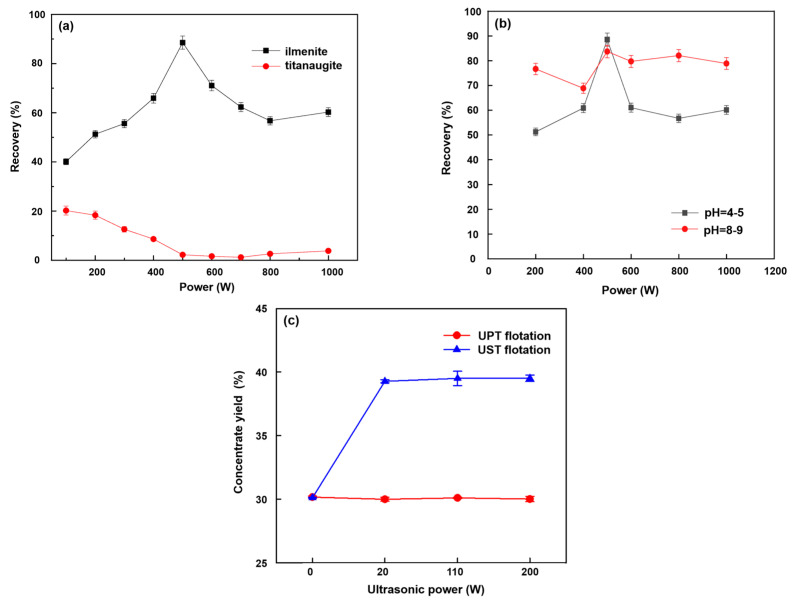
Effects of the kind of mineral (**a**) [96], the operating parameters (**b**) [95] and the ultrasonic treatment method (**c**) [94] on the mineral flotation performance.

Ultrasonic power describes the energy input in a certain ultrasonic system. Filippov et al. [50] assessed the ultrasonic flotation of coarse-grained material using four ultrasonic intensities at a frequency of 20 kHz, with integrated amplitude control of 10–100%. This study concluded that an increase in intensity of ultrasonic treatment resulted in a sharp reduction of 40% (from 82% to 42%) in the recovery of potassium chloride (Figure 10a). At the same time, the flotation of halite also decreased from 5.71% to 1.99%. This was due to a decrease in the stability of the bubble–particle aggregates [50]. Gungoren et al. [88] also found that the micro-flotation recoveries tended to decrease at higher ultrasonic powers. It can thus be concluded that high power ultrasonic treatment had unfavorable effects on flotation [89,90]. However, in literature, there are several studies showing that the mineral flotation recovery rises and then falls with an increase in ultrasonic power. For instance, Gungoren et al. [89] reported that the micro-flotation recovery increased to 63.64% and 65.57% with the use of ultrasonic treatment at 30 W and 90 W; then, it decreased to 37.50% at 150 W ultrasonic power (Figure 10b). Cao et al. [91] showed that the oxidized pyrite recovery increased gradually with the increase in ultrasonic power, and reached a maximum (89.32%) at 70 W. In this ultrasonic power range, the flotation performance of pyrite was improved due to the removal of oxidation products from its surface via the cleaning effect of an ultrasonic wave. However, the recovery was relatively lower at 90 W/100 W compared to that at 70 W. The reasons were the following: Firstly, the surfaces of these pyrite particles were oxidized in the ultrasonic field of 90 W/100 W due to the longer time involved [91]. Secondly, the stronger the ultrasonic intensity at 90 W/100 W, the greater the turbulence, which was not conducive to the adhesion of bubbles and particles [92]. In addition, surface defects on the surface of pyrite were introduced by stronger ultrasonic power, leading to a reduction in the pyrite surface hydrophobicity [91]. However, others take the opposite view. Altun et al. [93] reported that the combustible recovery of Himmetoğlu oil shale decreased and then increased with an increase in ultrasonic power. Beyond that, the kinds of minerals, operating parameters, and ultrasonic treatment methods (such as ultrasonic pretreatment (UPT) flotation and ultrasonic simultaneous treatment (UST) flotation) had different effects on mineral flotation performance with the same ultrasonic power (Figure 11) [94,95,96].

### 3.2. Ultrasonic Frequency

**Figure 12:**
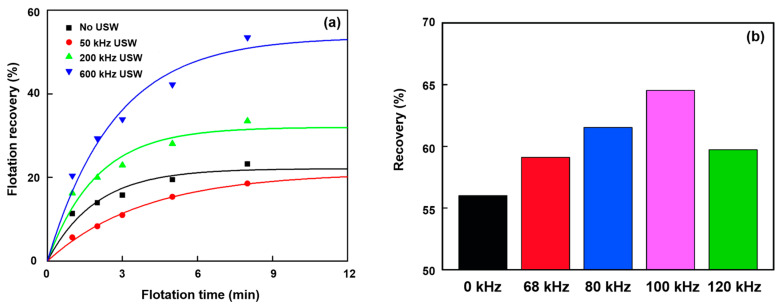
Influences of the flotation time (**a**) [98] and the ultrasonic frequencies (**b**) [101] on flotation effects.

Ultrasonic frequency is defined as the number of periodic oscillations per second, usually expressed in Hertz (Hz). Commonly, the critical size of a cavitation bubble is determined via ultrasonic frequency [97]. Various ultrasonic frequencies lead to different mineral flotation results. Chen et al. [98] reported that the ultimate recoveries were 21.12, 32.02, and 53.36% at ultrasonic frequencies of 50 kHz, 200 kHz, and 600 kHz, respectively. There was an increase in ultimate recovery with an increasing ultrasonic frequency (Figure 12a). Similar results were reported by Li et al. [99]. However, a reported effect of the ultrasonic frequency on bauxite flotation was inconsistent [98]. Ouyang et al. [100] studied the use of ultrasonic treatment for the desulfurization effect in bauxite flotation and found that the desulfurization effect of bauxite pulp treated with a frequency of 20 kHz was better than that of 60.6 kHz. In addition, Wang et al. [101] found that the floatability of fine coal slimes increased with an increase in the ultrasonic frequency from 0 kHz to up to an optimum value of 100 kHz, beyond which the floatability decreased at a higher ultrasonic frequency (Figure 12b). Lu et al. [102] also drew a similar conclusion after testing out oxidized pyrrhotite flotation using ultrasonic treatment with an ultrasonic frequency in the range of 20–60 kHz.

## 4. Effect of Ultrasonic Treatment on Mineral Flotation

Ultrasonic effects on flotation can be summarized as ultrasonic effects on minerals, flotation agents, and pulp, which are based on the ultrasonic cavitation effect and acoustic radiation force effect.

### 4.1. Effect of Ultrasonic Treatment on Minerals

The effect of ultrasonic treatment on minerals mainly results from the mechanical agitation generated by the cavitation effect of ultrasonic treatment, which is mainly carried out in the pretreatment of minerals. The huge energy released by the instantaneous collapse of cavitation bubbles in the ultrasonic field can clean, dissolve, and desulfurize the mineral surface.

#### 4.1.1. Cleaning Effect

**Figure 13:**
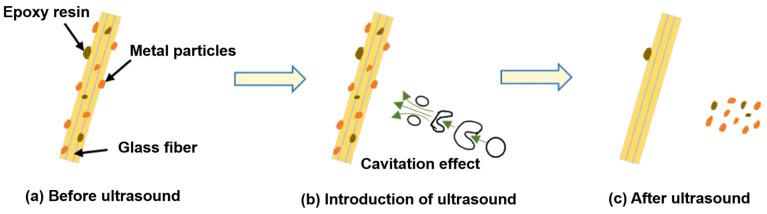
Mechanism of ultrasound–glass fiber interaction [106].

Mineral surface impurities can be removed by ore washing, that is, slime, slime coatings, and oxide films covering a mineral surface can be removed under the action of hydraulic and mechanical forces [103,104]. The cavitation of an ultrasonic wave in water can be used to clean the mineral surface, as shown in Figure 13. The shock wave emitted by bubble transient cavitation produces a pressure thousands of times that of atmospheric pressure around the mineral and repeatedly impacts the impurity layer on the mineral surface, thus destroying the adsorption between the impurity layer and the mineral surface and causing the impurity layer itself to break away from the mineral surface. The stable cavitation of bubbles can also scrub the surface, and tiny bubbles “drill” into mineral cracks to vibrate them and cause impurities to fall away [105].

**Figure 14:**
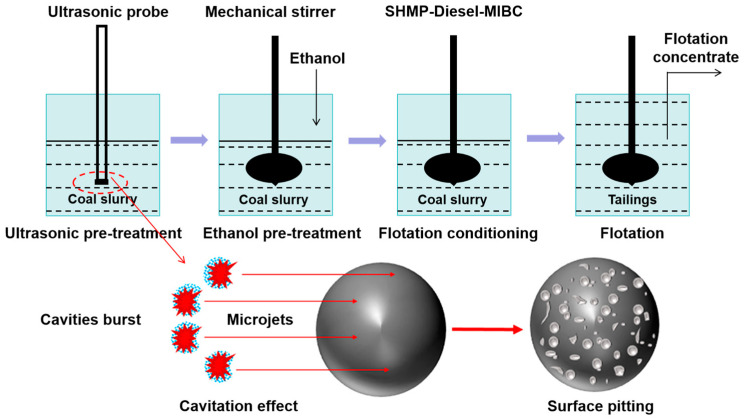
Schematic illustration of the effect of ultrasound–ethanol pretreatment on the floatability of oxidized coal [109].

**Figure 15:**
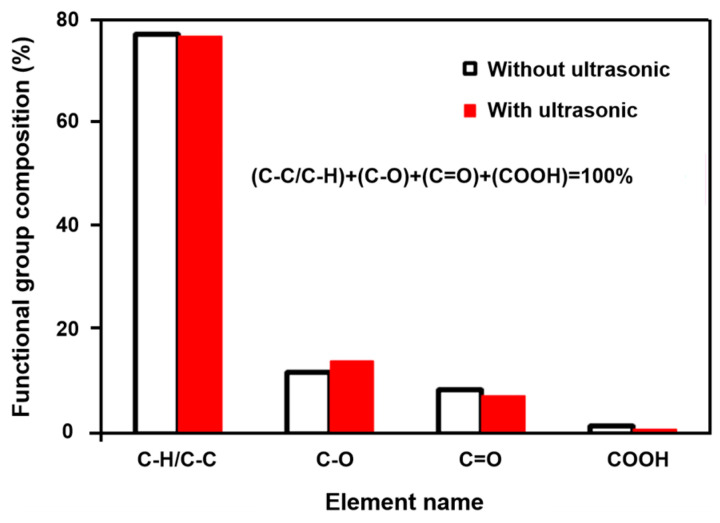
Composition of functional groups on lignite surfaces with/without ultrasonic treatment [111].

There has been much research on mineral surface cleaning through ultrasonic treatment. Ozkan et al. [107] carried out coal flotation in ultrasonically assisted flotation cells and found that the use of ultrasonic treatment to clean the surface of coal particles may be beneficial due to the thorough contact between the collector and the coal particles, thus greatly reducing the consumption of reagents and improving the recovery of coal flotation. Therefore, more positive results may be obtained with ultrasonic energy inputted into a flotation system than with conventional coal flotation. Gungoren et al. [88] investigated the improvement possibilities for the floatability of galena in the presence of ultrasonic application. It was found that the improvement of the recovery rate of micro-flotation is related to the enhancement of galena hydrophobicity by ultrasonic treatment. The results showed that the maximum recovery rate of micro-flotation reached 77.5% with 30 W of ultrasonic power. The enhancement of galena hydrophobicity by low-power ultrasonic treatment can be attributed to the increase in fresh galena surface, thus improving the adsorption efficiency of the collector. Gungoren et al. [89] found that the quartz-amine flotation recovery increased from 45.45% to 63.64% with 30 W ultrasonic application. Cao et al. [91] studied the influence of an ultrasonic wave on the surface properties of oxidized pyrite, and found that the ultrasonic wave had cleaning and oxidation effects on the surface, which could remove impurities attached to the surface. Hassani et al. [108] used ultrasonic irradiation as a pretreatment method to reverse the flotation of phosphates, and they found that the efficiency of phosphate flotation when using ultrasonic irradiation was higher compared to that of conventional flotation. This is because cavitation bubbles cleaned the surfaces of particles. When cleaner, the final grade and recovery of P_2_O_5_ reached 21.31% and 77.18%, respectively. Barma et al. [109] found that ultrasonic pretreatment can reduce the content of hydrophilic oxygenated functionalities on the surface of coal and improve the hydrophobicity of coal, thus significantly improving its floatability. A maximum yield and combustible matter recovery of 65.92% and 87.55%, respectively, were obtained under combined ultrasound–ethanol pretreatment in the flotation concentrate. A schematic illustration showing the ultrasound–ethanol pretreatment effect during the oxidized coal flotation process is displayed in Figure 14. Shi and Shi found the same results [110]. They reported that the aromatics (CH, –CH), phenols, alcohols, ethers, and ester C-O group on the coal surface increased, while some oxygen-containing groups disappeared after ultrasonic treatment, resulting in enhanced hydrophobicity on the coal surface. Thus, the recovery rate of fuel and the flotation perfection index after ultrasonic treatment are significantly improved. However, Peng et al. [111] reported that functional groups on lignite’s surface, including C-C/C-H, C-O, C=O, and COOH, underwent no apparent change after ultrasonic treatment, as presented in Figure 15. Videla et al. [56] treated copper sulfide tailings ultrasonically and found that the ultrasonic cavitation effect could clean the surfaces of mineral particles and reduce the adsorption of slime coating, thus promoting the action of agents. An improvement in copper recovery of up to 3.5% was obtained.

#### 4.1.2. Ultrasonic Corrosion

The surface dissolution of minerals affects the surface properties of minerals mainly by changing the quantity, state, and position of elements on the surfaces of minerals, which can be realized through physical or chemical methods [112]. The surface dissolution of minerals can cause a change in crystal chemistry, surface chemistry, and solution chemistry, thus affecting the flotation behavior of minerals. Surface dissolution as a pretreatment method to change the surface properties of minerals is helpful to improve the flotation behavior of those minerals. For example, when spodumene is pretreated with NaOH, dissolution occurs on the mineral surface, the relative contents of lithium and aluminum on the mineral surface increase, and the flotation recovery of spodumene increases [113]. In addition, Zheng et al. [114] found through research that NaOH soaking treatment can cause spodumene surface corrosion, and mechanical agitation can further promote the surface corrosion of spodumene and improve its floatability to a greater extent. As an energy form of ultrasonic treatment, mechanical agitation caused by the ultrasonic cavitation effect can achieve an effect that ordinary low-frequency agitation cannot, strengthen the selective dissolution effect of the mineral surface, and create favorable conditions for flotation.

**Figure 16:**
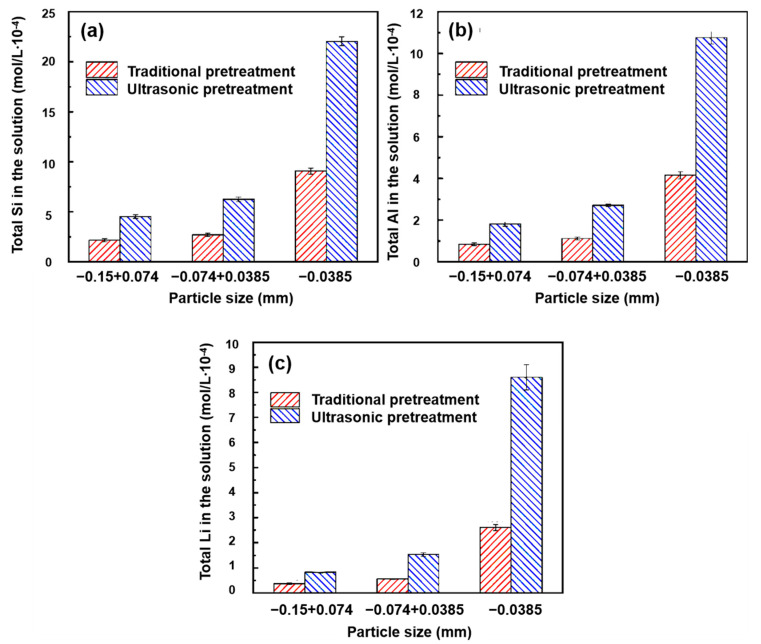
Concentration of main elements Si (**a**), Al (**b**) and Li (**c**) after pretreatment [118].

**Figure 17:**
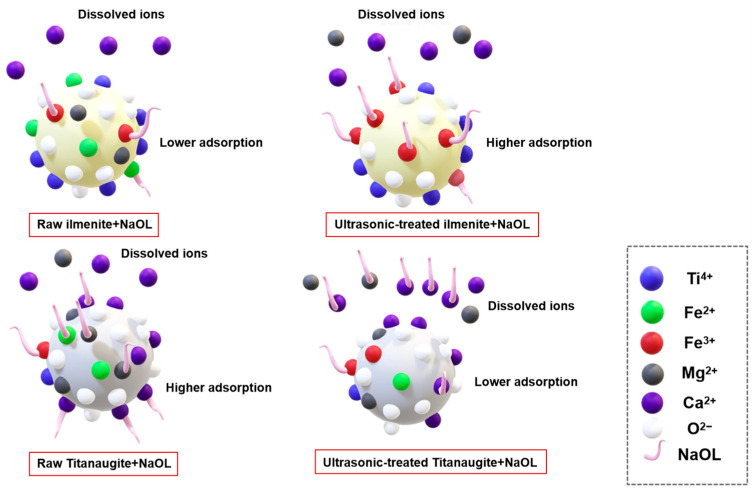
Adsorption model for ilmenite and titanaugite before and after ultrasonic treatment using NaOL as the collector [118].

Certain researchers [95,115,116,117] used ultrasonic treatment as a surface modification method, to explore its influence on mineral flotation. Chu et al. [118] found that Si species on the surfaces of coarse particles are easily dissolved into the solution, thus increasing the relative contents of Al and Li species on the surfaces in the presence of ultrasonic treatment, and then the selectivity of surface dissolution was slightly reduced. This was conducive to the adsorption of the collector on the surface. For spodumene particles smaller than 0.0385 mm, ultrasonic treatment will lead to a more serious reduction in solubility selectivity. Fortunately, the large amount of dissolution makes up for the loss caused by the decrease in selectivity. The concentration of the main elements after pretreatment is shown in Figure 16a–c. Wu et al. [96] reported that Fe^2+^ ions on the surface of ilmenite were oxidized to Fe^3+^, which increased the adsorption of NaOL on the surface of ilmenite, while Ca^2+^ and Mg^2+^ were exposed and dissolved in solution, which inhibited the adsorption of NaOL on the surface of ilmenite after ultrasonication. Therefore, separation between ilmenite and titanaugite can be realized more effectively. Figure 17 displays an adsorption model for ilmenite and titanaugite before and after ultrasonic treatment. Shu et al. [95] investigated the flotation and possible adsorption mechanisms of the ilmenite surface before and after ultrasonic pretreatment. It was found that ultrasonic treatment promoted the oxidation of Fe^2+^ to Fe^3+^ and the solubilization of Ca^2+^ and Mg^2+^ at pH 4~5. Under weak alkaline conditions, ultrasonic treatment can also lead Ca^2+^ and Mg^2+^ to readsorb onto the surface of ilmenite as the main active sites. Therefore, the promotion effect of sonication on ilmenite seems remarkable, according to flotation results, due to the change whereby metal ions become active sites on the ilmenite surface. Huang et al. [119] investigated the influence of ultrasonic pretreatment on scheelite surface dissolution. They found that accelerating the dissolution of WO_4_^2−^ and Ca^2+^ increased the Ca/W ratio and exposed the reactive site of Ca^2+^ on the mineral surface, which was conducive to interaction between sodium oleate and scheelite, thus improving the flotation recovery and flotation rate.

#### 4.1.3. Desulfuration

Ultrasonic desulfurization of minerals is mainly for coal. Coal will produce SO_2_ pollution in the process of combustion, so the removal of sulfur contents from coal has become an urgent task. Ultrasonic desulfurization is based on chemical effects caused by ultrasonic cavitation, that is, extreme effects such as s high temperature, high pressure, discharge, impact, and jet generated in liquid by ultrasonic cavitation are used to accelerate chemical reactions or realize reactions that are difficult to carry out under normal conditions [69]. Ultrasonic cavitation in an aqueous medium can induce the generation of free radicals (H^+^ and OH^−^), H_2_O_2_, and H_2_ (as shown in Equations (1)–(4)):

(1) $$H_{2}O_{2}\to {OH}^{-}+{OH}^{-}$$

(2) $$H^{+}+{OH}^{-}\to H_{2}O$$

(3) $$H^{+}+O_{2}^{-}\to {HO}_{2}$$

(4) $${HO}_{2}+{HO}_{2}\to H_{2}O_{2}+O_{2}$$

Under different pH conditions, these strong oxidants can oxidize with sulfur components on the surface of coal (see Equations (5)–(7)) and finally produce soluble sulfate in water.

(5) $$H_{2}S+H_{2}O_{2}\to S+2H_{2}O\;\mathrm{Acidic}\;\mathrm{pH}$$

(6) $${HS}^{-}+4H_{2}O_{2}\to SO_{4}^{2-}+4H_{2}O+H^{+}\;\mathrm{Neutrallty}\;\mathrm{pH}$$

(7) $$S^{2-}+4H_{2}O_{2}\to SO_{4}^{2-}+4H_{2}O\;\mathrm{Alkaline}\;\mathrm{pH}$$

Vasseghian et al. [120] studied the removal of ash and pyritic sulfur from bitumen using flotation with a low-frequency ultrasonic wave. Under the optimum conditions, the removal rates of pyrite sulfur (68.03% of total sulfur) and ash were 87.72% and 83.29%, respectively. Accordingly, the removal rates of ash and sulfur from pyrite by using column flotation in the ultrasonic mode were 11.9% and 10.3% higher than those without the ultrasonic mode, respectively. Significantly, however, the effect of ultrasonic treatment on the removal efficiency did not continue for more than 3 min. Zhang et al. [121] investigated the enhancement of desulfurizing flotation using the sonoelectrochemical method. The sulfur reduction reached up to 69.4%. The newly developed acoustic electrochemical enhanced flotation method realizes the combination of high sulfur reduction, high yield, and high ash content. Ultrasonic irradiation promotes electron transfer efficiency and increases the clean coal yield. Kang et al. [122] investigated the feasibility of improving desulfurization and de-ashing through ultrasonic treatment in the flotation process. They found that the output of clean coal after ultrasonic treatment increased by 19.53% while the sulfur and ash contents of cleaned coal decreased by 0.64% and 1.27%, respectively. The flotation perfect index was increased by 22.51%, the desulfurization perfect index by 25.36%, and the desulfurization rate by 2.49%. Therefore, it seems that ultrasonic treatment can improve the sulfur and ash removal in coal flotation. Kang et al. [123] studied the use of ultrasonic conditioning for the enhancement of the degree of desulfurization and flotation perfection by changing the pulp nature. Zhang et al. [124] used a sonoelectrochemical approach for the improvement of desulfurizing flotation of high-sulfur coal. A proportion of the total sulfur was removed using this method due to an increase in liquid–solid interface areas between coal particles and the solvent, thus improving mass transfer and then helping to remove sulfur. In addition, ultrasonic cavitation [125,126] destroys the weak bond of organic sulfide in coal particles and generates sulfide free radicals [127]. These free radicals combine with an organic solvent and then dissolve into a solution.

### 4.2. Effect of Ultrasonic Treatment on Flotation Agents

The mechanical agitation, mutual diffusion, and homogenization caused by ultrasonic cavitation will affect the flotation reagents. The dissolution, diffusion, and emulsification effects of flotation reagents can be enhanced by using these ultrasonic effects. Meanwhile, the properties of the solution and the aggregation state of the reagents will be changed.

#### 4.2.1. Dispersion and Emulsification

The mechanical effect, thermal effect, and cavitation phenomenon produced by an ultrasonic wave in pulp increase the pressure and temperature in the pulp. A local strong disturbance effect leads pharmaceutical molecules to dissociate rapidly under the strong pressure, while the ultrasonic acoustic flow effect has a stirring effect at the macro level, which increases the speed of circulation of the pharmaceutical and its dispersion rate [128]. In this way, ultrasonic treatment can promote the dispersion and emulsification processes of the reagent as a whole.

Ultrasonic treatment of flotation reagents can reduce the dosage of reagents, reduce their action time, simplify the flotation process, improve the flotation index, and bring certain economic benefits, so there are a lot of studies on the emulsification and dispersion of flotation reagents through ultrasonic treatment. Chen et al. [127] studied the influence of ultrasonic treatment on the dispersion of the oil collector in coking coal flotation slurry. They found that diesel oil disperses more evenly and stably in water after ultrasonic treatment. This is due to the strong mechanical forces on the oil–water interface, such as shock waves and micro-jets generated by ultrasonic cavitation, which break the oil droplets into fine oil droplets and promote their emulsification. Compared with mechanical agitation (7 µm), the average diameter of oil droplets in the presence of ultrasonic treatment largely decreased (3 µm). In addition, the energy produced by ultrasonic waves acted uniformly on the oil–water interface to maintain the stability of a diesel oil dispersion in water. Collector consumption may reduce as a result of ultrasonic treatment [129]. Burov et al. [130] investigated the effect of foam-forming compositions of flotation reagents, such as hydro-chloric amine solution, hydrochloric amine solution, and hydrochloric amine solution, on the properties of two-phase foams under ultrasonic treatment. They found that ultrasonic treatment enhanced the stability of the foam layer (in the case of the hydrochloric amine solution). Amine floccules were more efficiently distributed over the entire emulsion due to ultrasonic dispersion. Huang et al. [131] studied the effect of ultrasonic treatment on the solution properties and microstructure of benzohydroxamic acid (BHA) in order to separate scheelite from calcite effectively. The results showed that ultrasonic treatment can disperse the agglomeration structure of BHA and form smaller or single molecular structures, which reduces the steric hindrance effect between BHA molecules and minerals. All of these promoted the formation of Pb-BHA complexes between activated Pb^2+^ ions and BHA on the scheelite surface, increasing the adsorption heat of the interaction between BHA and scheelite to increase the adsorption rate of BHA on the scheelite surface. Ozkan [132] reported that the consumption of reagents drastically decreased with ultrasonic treatment due to the full contact between the collector and the coal particles. Chen et al. [133] investigated the mechanism of action of ultrasonic treatment on flotation reagents, such as xanthate aerofloat, calcium hydroxide, and oleic acid. They found that ultrasonic treatment accelerated the dissolution and diffusion of flotation reagents and it had a dispersive emulsification effect on slightly soluble agents. Chen et al. [133] studied the mechanism of action of ultrasonic waves on common flotation reagents by dyeing them. In this way, the dispersion rate was observed by pigment diffusion, and the emulsification effect was determined by the change in gray level. The results showed that ultrasonic treatment can be used in various pieces of flotation equipment to accelerate dispersion and emulsification. Flotation decarburization of fly ash assisted by an ultrasonic wave was studied by Li et al. [134]. The flotation efficiency, burn loss rate, and recovery rate of the concentrate were improved by adding the ultrasonic dispersing and emulsifying collector kerosene in a flotation test. He [135] conducted a combined modification test where they investigated the chemical modification and ultrasonic emulsification of a foaming agent. When using the solubility, surface tension, and flotation effect as indexes, the results showed that ultrasonic emulsification could improve the efficiency of the foaming agent and the bubble performance.

#### 4.2.2. Change in Properties and Microstructure of Pharmaceutical Solution

The collapse of cavitation bubbles will be accompanied by extreme effects such as high temperature, high pressure, discharge, and shock waves, resulting in various physical and chemical reactions of flotation reagents in solution. These reactions change the structure of some reagent molecules, for instance, inducing the formation of polar ions or polar states and destroying the balance of action between molecules, so that hydration weakens and the force is reduced. The original gas dissolved in the liquid escapes because of the supersaturation state, which leads to changes in the properties and microstructure of the flotation reagent solution such as conductivity, surface tension, pH, and so on.

##### Surface Tension

**Figure 18:**
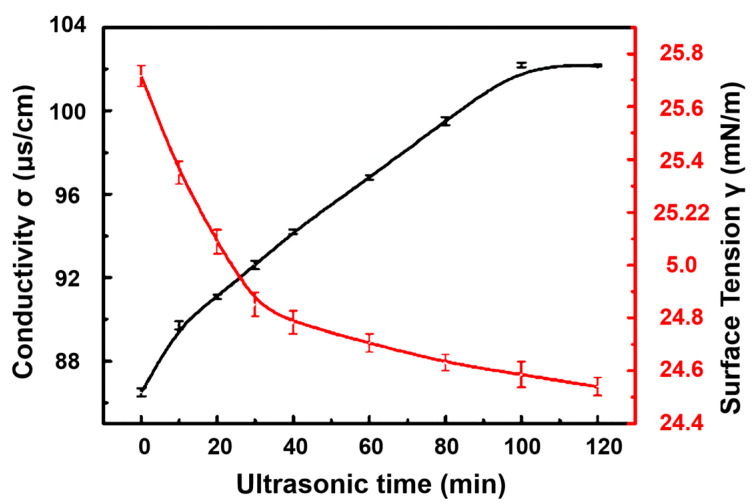
Role of ultrasonication in affecting the conductivity and surface tension of sodium oleate solution [119].

Burov et al. [130] studied the effect of foam-forming compositions of flotation reagents when using ultrasonic treatment on the properties of two-phase foams and found that the surface tension of foam-forming components was reduced after ultrasonic treatment, which was related to the cavitation dispersion of amine floccules, which were more effectively distributed on the surface of the emulsion. Ultrasonic treatment with a power of 420 W reduced the surface tension by 10%. Huang et al. [119] investigated the effects of ultrasonic pretreatment on scheelite surface dissolution, as well as the properties and microstructure of sodium oleate solution. With an increase in ultrasonic duration, the high temperature and high pressure during the ultrasonic process destroyed the intermolecular hydration layer, weakened the hydration effect, and caused the surface tension of sodium oleate solution to decrease rapidly and stabilize at 100 min (ultrasonic duration) (Figure 18). The same team [131] also studied the effect of ultrasonic treatment on the collector BHA and found similar results. Kang et al. [123] reported a decrease in the interfacial tension after ultrasonic conditioning, whereby ultrasonic treatment changed the internal structure of water and possibly broke some of its hydrogen bonds.

##### Conductivity

Huang et al. [119] reported that the conductivity of sodium oleate solution (NaOL) was improved via the cavitation effect of ultrasonic treatment. The conductivity of NaOL increased with the ultrasonic duration and power, ultimately reaching the equilibrium with an ultrasonic duration of 100 min and ultrasonic power of 630 W. The number of oleate ions and sodium ions in the solution increased due to the destruction of the dissociation equilibrium of NaOL by the cavitation effect of ultrasonic treatment. The conductivity of NaOL increased with an increasing concentration, and it increased more slowly above 400 mg‧L^−1^, because dissociation of NaOL molecules was promoted at low concentrations and hindered at high concentrations. Their research group also studied the effect of ultrasonic treatment on the solution properties and microstructure of BHA in order to achieve effective separation of scheelite and calcite [131]. They obtained similar results. There was an increase in the conductivity of BHA with the increase in ultrasonic time and ultrasonic power. The reason for this was that ultrasonic cavitation destroys the hydrogen bond break in R-CO-NHOH, promotes its dissociation, increases the number of free ions in the solution, and increases the conductivity of the solution. Hassanzadeh et al. [136] found that liquid conductivity slightly changed at a short ultrasonication time (t < 1.5 min), and then it dramatically increased from 2.2 µs/m (1.5 min) to 4.9 µs/m (30 min). The conductivity of the electrolyte increased with an increase in ion concentration in connection with a change in pH and the creation of highly reactive radicals (·OH and ·H). In addition, ultrasonic cavitation accelerated the surface oxide dissolution of the mineral. Thus, the solution conductivity increased. Li et al. [99] reported that the electrical conductivity of deionized water, calcium silicate solution, and calcium oleate solution increased with an ultrasonic time extension, but the electrical conductivity increase in calcium oleate solution was obviously higher than that in deionized water. Meanwhile, the increase in the electrical conductivity of calcium oleate solution was close to that of the deionized water, indicating that the ultrasonic process can promote calcium silicate dissolution but has little effect on the solubility of calcium oleate. Lu et al. [102] found that the conductivity of pulp reached 60.0 μs/cm after ultrasonic treatment for 12 min. The ion concentration in the pulp increased due to the strong solubilizing effect of the ultrasonic wave on the surface components of pyrrhotite.

##### pH

Kang et al. [123] found that ultrasonic conditioning resulted in an increase in the pH value of pulp. In the ultrasonic cavitation process, nascent oxygen, ‧OH, and ‧H free radicals have very high activity. OH free radicals have powerful oxidization capabilities, and Fe^2+^ is oxidized to Fe^3+^. The oxidation equation can be seen Equation (8).

(8) $${Fe}^{2+}+OH={Fe}^{3+}+{OH}^{-}$$

The ‧OH radicals gain an electron and become OH^−^ ions, increasing the OH^−^ ion concentration and pH value of the pulp. The effect of ultrasonic treatment on the pH of pulp was investigated by Chen et al. [137]. The results revealed that the pH value of pulp increased with an increase in ultrasonic treatment time. Water can be decomposed into H^+^ and OH^−^ under ultrasonic irradiation, and H^+^ preferentially adsorbs on the mineral surface or promotes the reverse reaction of the generation of H_2_O_2_ and H^+^, as shown in Equations (9) and (10).

(9) $${Fe}^{2+}+OH={Fe}^{3+}+{OH}^{-}$$

(10) $$H_{2}O_{2}+H^{+}\to H_{2}O+{OH}^{-}$$

After the ultrasonic treatment of pulp, interaction between oxygen and the pyrite surface is promoted, so that the oxygen content in pulp tends to decrease and the concentration of OH^−^ ions increases [137]. In addition, the introduction of ultrasonic cavitation leads the pH value of the pulp to decrease. Hassanzadeh et al. studied the effect of ultrasonication on the floatability of minerals [136]. The results indicated that the average pH value was down from 7.8 to 6.4 after ultrasonic treatment for 30 min. Similar results have been reported by Giriûnienë and Garðka, with a decrease in the pH of DI water when using an ultrasonication device at 34 kHz due to an increase in the hydrogen ions’ concentration in the water with increasing time, then arriving at the equilibrium value [138]. Li et al. [99] investigated the influence of ultrasonic treatment on the floatability of calcite in a sodium silicate/sodium oleate system. They found that the pH value of pulp decreased with the increase in ultrasonic power and ultrasonic time. The reason was that an ultrasonic wave will produce H_2_O_2_ when acting on water, and the concentration of H^+^ in the solution will increase, leading to a decrease in pH. In agreement with the results reported previously, it was confirmed that the pH of pulp decreased with the increase in ultrasonic treatment time [102,139].

### 4.3. Effect of Ultrasonic Treatment on Slurry

The effect of ultrasonic treatment on pulp mainly refers to the use of bubbles in pulp, including the formation and coalescence of microbubbles. The pulp will be inflated and stirred continuously when entering the flotation machine, and it produces a large number of bubbles that suspend the ore particles. The behavior of bubbles in the pulp is the key to affecting the flotation index. The formation and movement of bubbles in pulp can be improved by adjusting the ultrasonic frequency and power rate, so as to improve flotation behavior.

#### 4.3.1. Formation of Microbubbles

**Figure 19:**
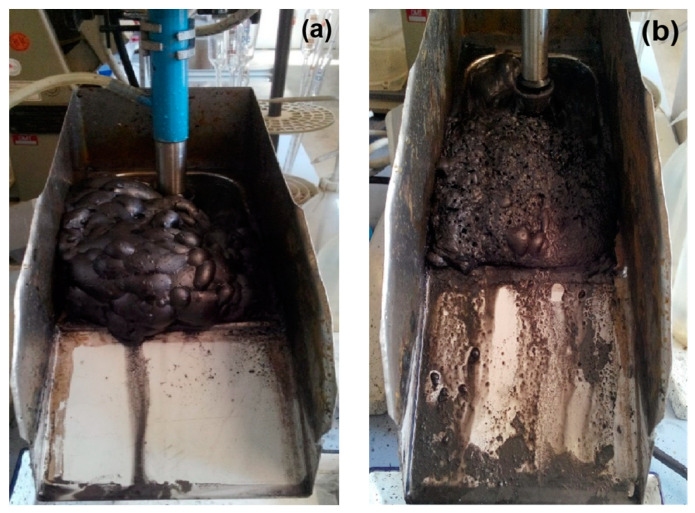
Photos of the coal froths: (**a**) conventional; (**b**) ultrasonic [140].

Bubble size has an important effect on mineral flotation, and fine bubbles are usually required [91,140]. The ultrasonic cavitation effect can cause the formation of tiny bubbles in the pulp (Figure 19) [91,111,141,142]. The formation of the small bubbles during ultrasonic treatment may be caused by two factors: (1) stable cavitation bubbles generated from the nuclei, and (2) conventional large bubbles bursting into small bubbles due to fragmentation [66].

**Figure 20:**
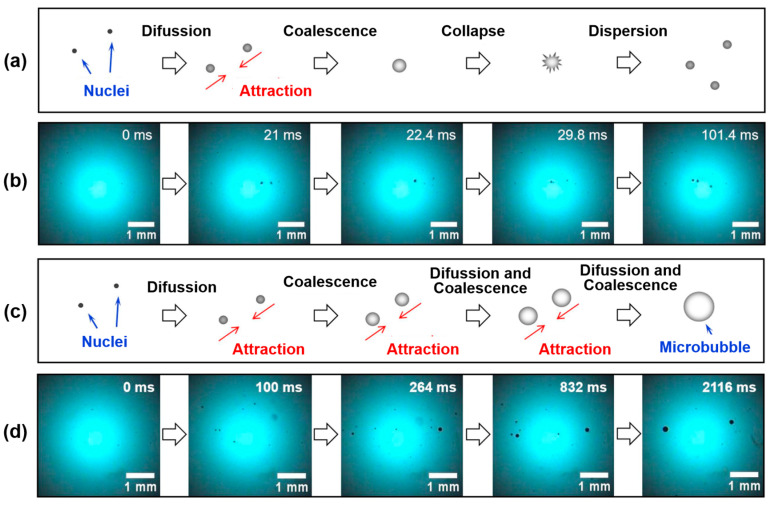
Microprocesses of bubble formation in 50 and 200 kHz USW fields at 0.25 Vpp ((**a**) bubble formation at 50 kHz; (**b**) images captured by a high-speed camera at 50 kHz; (**c**) bubble formation at 200 kHz; (**d**) images captured by a high-speed camera at 200 kHz) [98].

Cao et al. [91] investigated the effects of ultrasonication power on the flotation of an oxidized pyrite. The results showed that ultrasonication was conducive to pyrite flotation due to the formation of fine bubbles in the flotation cell. The mean sizes of bubbles were 2.55 mm, 2.01 mm, and 1.75 mm in water without ultrasonic treatment or treated with 20 W or 100 W of ultrasonic power, respectively. Chen et al. [98] studied the effect of the ultrasonic standing wave (USW) field with various frequencies on the fine coal flotation. Three sub-processes, the formation of microbubbles, the dispersion of conventional flotation bubbles, and the movement of particles during the attractive mineralization process, were analyzed by using a high-speed camera and focused beam reflection measurement (FBRM) (Figure 20). The results showed that the flotation metallurgical response was the largest at the highest USW frequency (600 kHz). Significantly, the flotation results of the low USW frequency (50 kHz) were even lower than those of conventional flotation tests. This was because of the effect of the frequency on carrier bubble formation and secondary acoustic force in the USW-assisted flotation process.

#### 4.3.2. Coalescence

**Figure 21:**
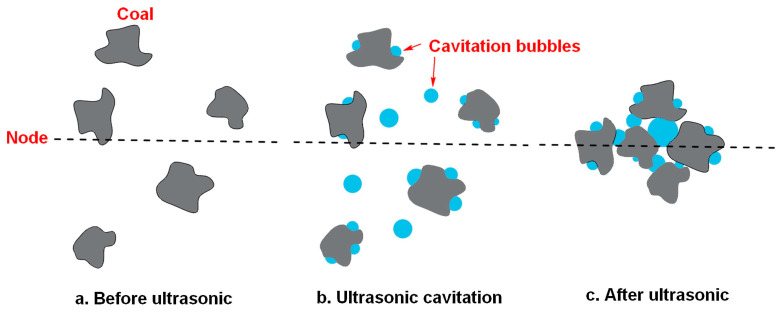
Mechanism of coal aggregation following the application of an ultrasonic standing wave [146].

Ultrasonic treatment is helpful in bubble coalescence, leading to coal coalescence. In recent years, the aggregation of particles, oils, or bubbles has been extensively studied [143,144,145]. Coalescence refers to the use of ultrasonic treatment to lead bubbles attached to fine minerals to aggregate, which increases flotation recovery. The bubble aggregates are like a tight “net” collecting the particles (Figure 21) [94]. The flotation recovery of fine coal particles is always difficult because of the low collision efficiency between bubbles and fine coal particles [146]. One way to improve the flotation recovery of fine coal is to agglomerate fine coal before flotation [146].

**Figure 22:**
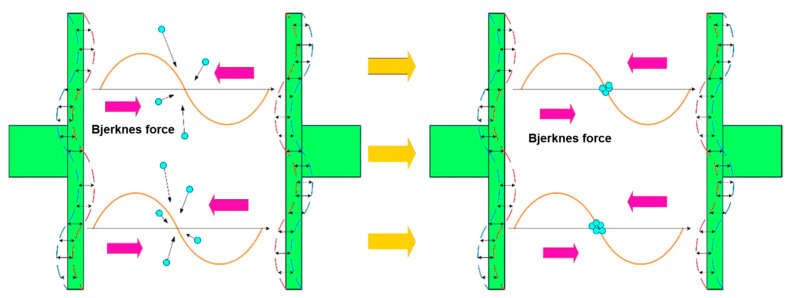
Mechanism of bubble aggregation in an ultrasonic field [148].

**Figure 23:**
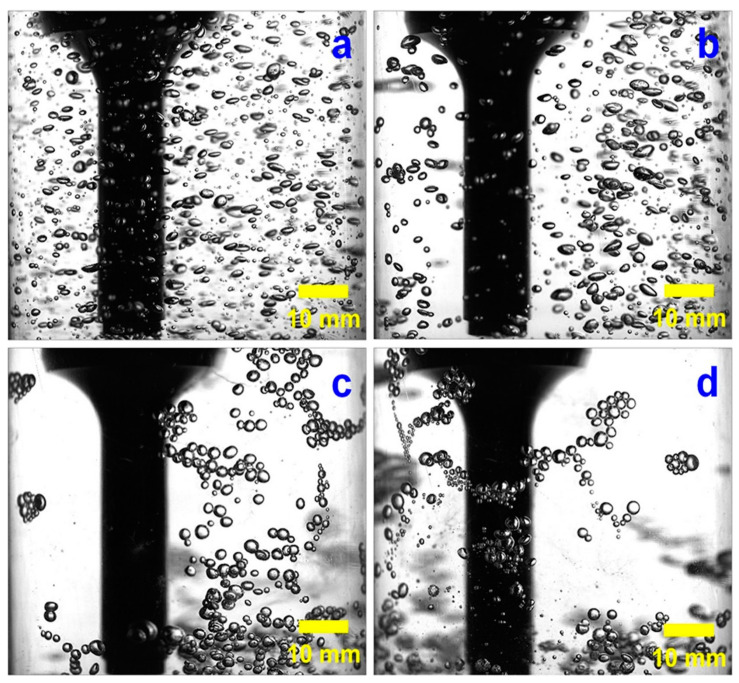
Images of the effects of ultrasonic power on bubble aggregates at 3 s of ultrasonic treatment ((**a**) 0 W; (**b**) 20 W; (**c**) 110 W; (**d**) 200 W) [94].

**Figure 24:**
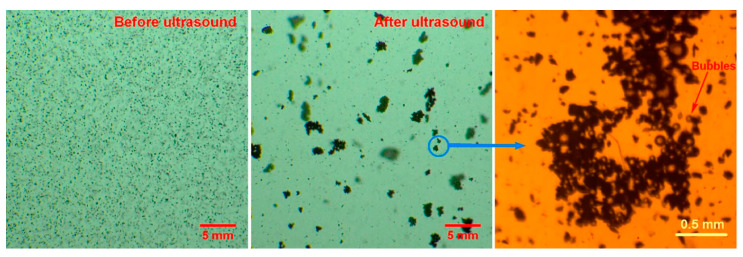
Aggregation of coal particles (74–125 μm) by USW at 200 kHz [66,146].

Cilek et al. [57] investigated the effects of ultrasonic treatment on froth and pulp phases in the flotation of barite and chalcopyrite samples, respectively. They found that the introduction of ultrasonic treatment accelerates bubbles’ coalescence, which may reduce the recovery of the foam phase in the ultrasonic flotation of barite and chalcopyrite. This is because the addition of ultrasonic treatment to the foam mainly affects the solid–liquid interface where cavitation occurs, thus speeding up the expulsion of particles in the water between the bubbles. This reduces the density and bubble viscosity between the pulp. Both of these phenomena lead to an increase in the bubble coalescence, leading to a decrease in the foam phase recovery. They also studied the influence of ultrasonic treatment on the flotation rate of a complex sulfide ore [147]. From the experimental results, it could be seen that there was a significant difference between the bubble size distributions of the flotation tests in the presence of and absence of ultrasonic treatment. A wider bubble size distribution was produced by adding ultrasonic treatment. Even if many bubbles coalesced, there were still a significant number of small bubbles. It was found that ultrasonic flotation was more effective with the shallow froths due to an increase in the bubble coalescence. Figure 22 illustrates the principle of bubble aggregation in an ultrasonic field [148]. Mao et al. [148] employed ultrasonic pretreatment (UPT) and simultaneous treatment (UST) with different ultrasonic powers to float coal. They found that the flotation of coal was improved when using ultrasonic treatment through the combined effect of cavitation and acoustic radiation force. The change in bubbles when using ultrasonic treatment was a reason for the enhancement in recovery. The aggregation and shapes of large bubbles were affected by ultrasonic treatment through the oscillation behavior and acoustic radiation force, respectively. Images of the bubble aggregation after 3 s of ultrasonic treatment at 0 W, 20 W, 110 W, and 200 W are presented in Figure 23. Wang et al. [149] investigated flotation bubble size distribution rules in the presence of ultrasonic treatment with various ultrasonic frequencies. The results showed that ultrasonic irradiation at a certain frequency can change the particle size of flotation bubbles and promote the agglomeration of bubbles. The average particle size of bubble flocs increased by 80~120 μm with 430 kHz of ultrasonic frequency. The second Bjerknes force between bubbles played an important role in the bubble aggregation. The effect of aeration velocity on bubble size and bubble aggregation was not significant. The aggregation of fine coal could be achieved via the manipulation of bubbles [146]. Chen et al. [146] studied the aggregation behavior of coal particles when adding an ultrasonic standing wave. Flotation tests of a 1 g/L coal particle (74~125 μm, 5% ash content) suspension were carried out under a 200 kHz ultrasonic standing wave. Figure 24 shows the coal aggregation recorded by a CCD camera and a microscope. When the size of the cavitation bubbles was larger than the resonance radius, the bubble-laden coal particles moved toward the ultrasonic standing wave through acoustic radiation force, resulting in the rapid aggregation of fine coal particles. Then, the flotation rate markedly improved. The flotation recovery increased from 57% to 68% at a 2 min flotation time.

## 5. Conclusions and Outlook

This paper has presented an overview of the ultrasonic-assisted flotation process for minerals. The positive effects of ultrasonic-assisted flotation of minerals are usually attributed to the transient cavitation effect, stable cavitation effect, and acoustic radiation force effect. The effects of the main ultrasonic parameters (ultrasonic power and ultrasonic frequency) on mineral flotation have been discussed. Most importantly, the applications of ultrasonic treatment in mineral flotation have been reviewed. The use of ultrasound technology is promising for the improvement of flotation performance. However, the application of ultrasonic treatment in mineral flotation is still in the research stage. So, future directions for research into ultrasonic treatment are proposed.

(1) The different effects of ultrasonic treatment on flotation are realized by precisely controlling the parameter conditions. Different parameter conditions may have different or even opposite effects on the flotation system, such as the dispersion or aggregation of mineral or bubbles. The processing of minerals under different conditions requires different effects of ultrasonic treatment. For example, in fine flotation, it is hoped that particles will be more easily adhered to the flotation bubble through agglomeration, so the conditions required in ultrasonic treatment to lead mineral particles to coalescence should be controlled well. Furthermore, it is hoped that bubbles’ agglomeration will be decreased and the collision probability between mineral particles and bubbles will be increased, so, here, the conditions for the formation of microbubbles by ultrasonic treatment should be controlled. However, at present, there is still a gap in the research into the effect of ultrasonic treatment on mineral flotation performance, which we propose should be a focus of future research.
(2) Until now, the applied research on ultrasonic treatment in mineral flotation has mostly been carried out on a laboratory scale, which may provide a theoretical basis for the future industrial applications of ultrasonic treatment. However, when ultrasound is applied on a commercial scale, this will involve very large scale-up ratios and a high degree of uncertainty. In order to effectively utilize this technology and start ultrasonic-based coal beneficiation treatment in practical operations, large-scale pilot studies are very important, which will help to determine amplification parameters and develop suitable ultrasonic-based equipment or reactors. In addition, the high energy loss and safety problems of ultrasonic treatment should be considered and resolved before industrial applications.
(3) Consideration is required of how industrial ultrasonic equipment typically operates at high powers, leading to intense corrosion caused by cavitation. This corrosion cannot be sustained by pipelines and building materials over an extended period, resulting in escalating costs. Furthermore, the operation of industrial ultrasonic equipment generates substantial noise and strong sound waves, which are detrimental to employee well-being and production safety.
(4) Appropriate pretreatment can eliminate the oxide layer on particle surfaces, enhance agent adsorption onto hydrophobic surfaces, and achieve surface hydrophobic modification of fine oxidized coal. The selection of pretreatment methods should consider factors such as the coal slime oxidation degree, feasibility at scale, and power consumption. Additionally, if ultrasonic or other pretreatment methods are combined organically, the flotation performance of fine oxidized coal can be further improved.
(5) Most studies have focused on coal grinding in specific regions, limiting the universality of the process. To enhance its applicability, it is recommended that coal grinding should be investigated across different regions. By examining the variations in coal grinding behavior during ultrasonic flotation, the universality of the process can be further improved.
